# Relative effect size-based profiles as an alternative to differentiation analysis in multi-species single-cell transcriptional studies

**DOI:** 10.1371/journal.pone.0305874

**Published:** 2024-06-25

**Authors:** Anna Papiez, Jonathan Pioch, Hans-Joachim Mollenkopf, Björn Corleis, Anca Dorhoi, Joanna Polanska

**Affiliations:** 1 Department of Data Science and Engineering, Silesian University of Technology, Gliwice, Poland; 2 Institute of Immunology, Friedrich Loeffler Institute, Greifswald, Germany; 3 Department of Immunology, Max Planck Institute for Infection Biology, Berlin, Germany; UT Health San Antonio: The University of Texas Health Science Center at San Antonio, UNITED STATES

## Abstract

Combining data from experiments on multispecies studies provides invaluable contributions to the understanding of basic disease mechanisms and pathophysiology of pathogens crossing species boundaries. The task of multispecies gene expression analysis, however, is often challenging given annotation inconsistencies and in cases of small sample sizes due to bias caused by batch effects. In this work we aim to demonstrate that an alternative approach to standard differential expression analysis in single cell RNA-sequencing (scRNA-seq) based on effect size profiles is suitable for the fusion of data from small samples and multiple organisms. The analysis pipeline is based on effect size metric profiles of samples in specific cell clusters. The effect size substitutes standard differentiation analyses based on p-values and profiles identified based on these effect size metrics serve as a tool to link cell type clusters between the studied organisms. The algorithms were tested on published scRNA-seq data sets derived from several species and subsequently validated on own data from human and bovine peripheral blood mononuclear cells stimulated with *Mycobacterium tuberculosis*. Correlation of the effect size profiles between clusters allowed for the linkage of human and bovine cell types. Moreover, effect size ratios were used to identify differentially regulated genes in control and stimulated samples. The genes identified through effect size profiling were confirmed experimentally using qPCR. We demonstrate that in situations where batch effects dominate cell type variation in single cell small sample size multispecies studies, effect size profiling is a valid alternative to traditional statistical inference techniques.

## Introduction

In the constantly developing fields of molecular biology and medicine, comparative multi-species studies are invaluable for advancing knowledge about basic biological processes or for guiding design of novel interventions. Species-specific reagents for non-model organisms, which often are sources of pathogen spill overs, are limited and in such instances agnostic investigations with a focus on transcript abundancies and transcriptional activity are feasible. Studying immune responses to stimuli across different species at single cell resolution offers unprecedented insights into host’s immune reactivity. Single cell RNA sequencing (scRNA-seq) has revolutionized the scene of high-throughput technologies. Profiling of single cells has indisputable advantages, e.g., revealing complex and rare cell populations, uncovering regulatory relationships between genes, and tracking the trajectories of distinct cell lineages in development [1]. Nevertheless, as with any high-dimensional data generation technique, it is not free of challenges during the analysis stages [2]. At the advent of rapidly growing databases with data sets freely available, one of the means to improve information retrieval efficiency is to integrate and combine data within and across different species and experiments. The reuse potential could yet be confined by batch effects as sources of bias. Thus, data must be handled with care to avoid false discoveries and incorrect conclusions. In many instances, the lack of sufficient sample sizes poses difficulties in efficient batch effect correction [3, 4]. When performing comparative studies of differential expression, standard statistical techniques or approaches such as p-value integration are biased. Batch effects often overpower the biologically driven variation, disrupting differential gene expression analysis. To address the batch effect correction in scRNA-seq data a variety of tools have been developed. Common techniques introduce corrections at the dimensionality reduction level and affect the embeddings to facilitate clustering tasks. For instance, canonical correlation analysis (CCA) implemented in Seurat [5] identifies shared correlation structures across data sets, and aligns these dimensions using dynamic time warping, yielding modified projection components. Harmony [6] returns normalized feature reduction vectors through iterative clustering. FastMNN [7] provides normalized principal components with the use of the mutual nearest neighbor method. These algorithms, however, do not affect the original expression matrix and do not mitigate the issue of batch effects impacting differential gene expression analysis. State-of-the-art tools for batch effect handling of microarrays and bulk RNA-seq data sets offer the possibility of obtaining a corrected gene expression matrix. Limma [8] may be used as a solution for filtering batch effects with a linear model. SVA [9] identifies and removes batch effects through latent factors (surrogate variables). ComBat [10] may be applied to adjust the expression matrix through empirical Bayes models, where the batch structure is known. Albeit successfully aiding the process of cell type identification by means of dimensionality reduction and clustering, these algorithms do not fully overcome the issue of batch effect driven variation. Other methods rely on normalization of gene expression matrices with regard to genes that are stably expressed across samples and conditions. scMerge [11] is an algorithm derived from the Remove Unwanted Variation III (RUVIII) model based on stably expressed genes and ZINB-WaVE [12] and applies a zero-inflated negative binomial model extension for the RUV model. These techniques are useful yet limited in their utility to data from model organisms, e.g., human or mouse genomes, and have sets of stably expressed genes identified. For comparative analysis of non-standard species, there is a need to resort to other methods.

**Fig 1:**
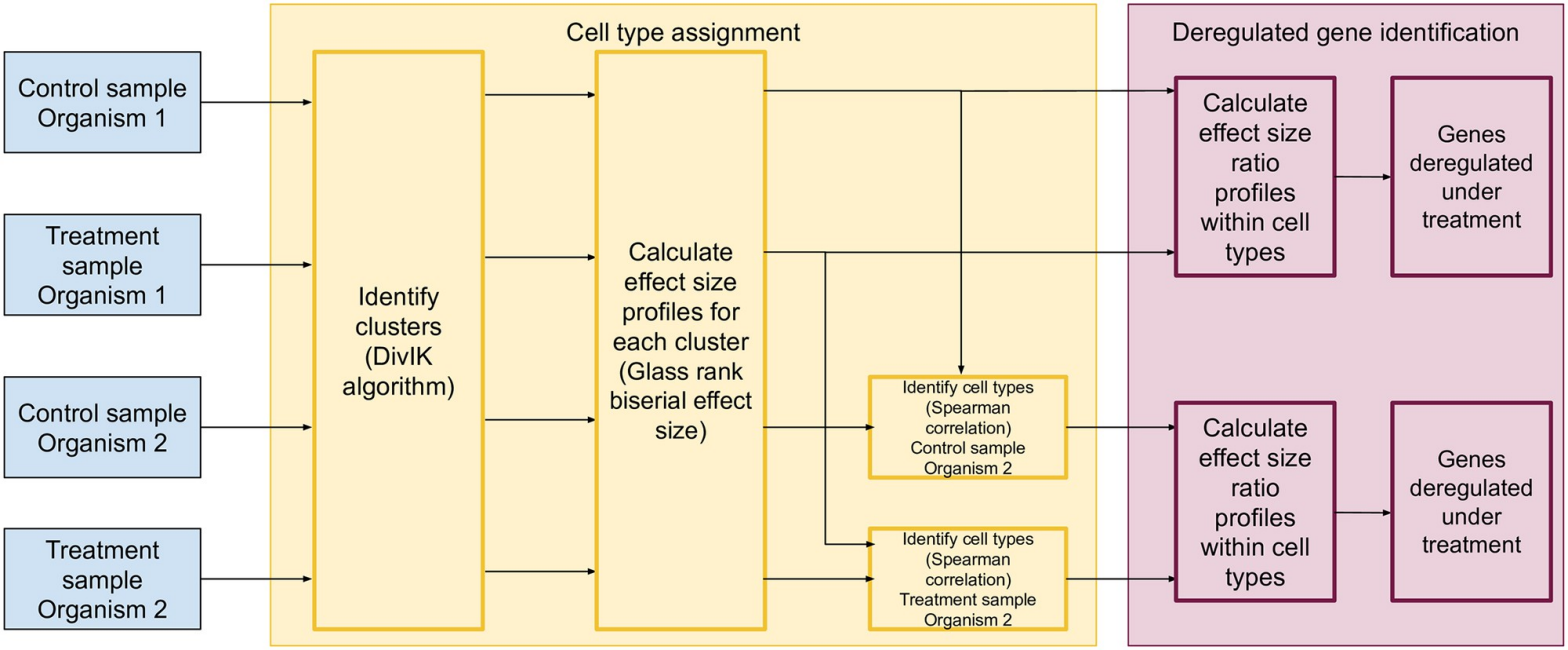
Diagram of the analysis workflow.

Here, we aimed to devise a pipeline enabling accurate analysis of small size datasets from non-model organisms. Using published and newly generated scRNA-seq data sets across various species we recognized that batch effects dominated cell subtype variation when combining experiments and that consistent clustering was a complex task. We employed effect size-based profiling, a novel approach in the context of scRNA-seq data, and report that this algorithm may be used as an alternative to classic statistical analyses of differentiation in gene expression for analysis of combined multi-species scRNA-seq studies. The analysis workflow is presented in Fig 1.

## Materials and methods

The samples analyzed in this study consist of human and bovine PBMCs, each with one control and one sample stimulated with lysate of *M. tuberculosis* (Mtb). Studies involving human blood samples were approved by the Ethics Committee of the University Hospital Greifswald under the approval numbers BB 184/17 and BB 114/19. The studies were approved in December 2017 and September 2019, respectively. The samples for the scRNA-seq experiments were collected in March 2019 under BB 184/17. All human samples for qPCR analysis were collected between October 2019 and December 2021 under BB 114/19. For collection of human blood samples written consent was obtained before sample collection. The bovine blood was collected and handled according to approvals granted from the state authority “Landesamt für Landwirtschaft, Lebensmittelsicherheit und Fischerei Mecklenburg-Vorpommern” (LALLF) (reference number LALLF 7221.3–2-041/17).

### Collection and processing

PBMCs sample from cattle and human were isolated using pancoll (Pan-Biotech) gradient density centrifugation following the manufacturer’s protocol. Purified PBMCs were counted using a Neubauer chamber and resuspended in RPMI-1640 + 10% FCS at a concentration of 1—5*x*10^6^ PBMCs/ml medium for downstream applications. Cells were rested overnight at 37°C and 5% CO2 before stimulation with 10 μg/ml Mtb H37Rv whole cell lysate (BEI Resources Repository NIAID) for 3h at 37°C and 5% *CO*_2_. Control samples were left untreated. For qPCR CD14+ monocytes were isolated using an anti-human CD14 antibody (Biolegend, Clone: M5E2) or an anti-bovine CD14 antibody (Kingfisher Biotech, Clone: CAM66A) using magnetic-based enrichment.

### Quantitative real time PCR

RNA was isolated by TRIzol (ThermoFisher) and chloroform extraction, washed with 75% ethanol and resuspended in RNAse-free *H*_2_*O* (Agilent). The RNA concentration was measured using a Nanodrop und stored at -80°C at a concentration of 100 ng/μl or used immediately for downstream procedures. 100 ng/μl RNA was transcribed into cDNA using the High-Capacity cDNA Reverse Transcription Kit (Applied Biosystems) following the manufacturer’s protocol. The PowerUp^™^SYBR^™^Green Master Mix (Applied Biosystems) was used for quantification of target genes by RT-PCR using 50 ng cDNA and 10μM primer in a total volume of 10 μl per reaction. MicroAmp^™^Optical 96-Well Reaction plates (Applied Biosystems) were sealed using MicroAmp^™^Optical Adhesive Film (Applied Biosystems) and loaded on a QuantStudio 6 Flex real-time PCR cycler (ThermoFisher). Samples were run in triplicates and a *H*_2_*O* negative control for each condition and primer pair. Primer sequences and sources are provided in S1 Table. Primer pairs were validated before use. For this, efficiencies were evaluated and required compatibility between human and bovine samples. In addition, primer pairs had to generate a single product and show a single respective melt curve analysis. All genes of interest were normalized against expression of GAPDH and RPS13 and the average fold change was reported.

### scRNA-seq experiment

For scRNA-seq, samples were washed with 9 ml of PBS (Gibco)+0.04% BSA at 400xg for 10min. Cells were resuspended in 1ml PBS+0.04% BSA and counted using a Neubauer chamber. 1*x*10^6^ PBMCs from each sample were transferred into a new 15ml polypropylene tube and filtered using a 70μm cell strainer (FisherScientific) before adjusting the cell concentration to 1*x*10^6^ PBMCs/ml PBS + 0.04% BSA. Cells were loaded on the 10x genomics to achieve analysis of 10,000 cells per sample by using the Chromium single cell 3’ library V2 (10x genomics) and following the manufacturer’s instructions. Library quality and concentration was assessed by Bioanalyzer (Agilent Technology) and the KAPPA library quantification kit (Roche) following the manufacturer’s instructions. Libraries were pooled and sequenced using a on an Illumina HiSeq1500 instrument using paired-end sequencing (26 bp Read 1 and 98 bp Read 2) with a single sample index (8bp i7 index) and using a HiSeq Rapid SBS Kit V2 (Illumina).

### Data preprocessing

Raw sequencing data were preprocessed with the Cell Ranger software [13]. The filtered gene-barcode matrixes were used for further calculations. In each sample features with no detected cells were discarded and cells with feature count greater than 200 and percentage of mitochondrial DNA below 10 were retained. These data were log-normalized with a scale factor of 10,000 and scaled and centered before clustering.

### Clustering

Standard algorithms applied nowadays for clustering of single cell data include approaches based on hierarchical clustering (SINCERA [14], BackSPIN [15], CIDR [16]), k-means (SC3 [17], RaceID [18], SIMLR [19]), graph-based techniques (scanpy [20], Seurat [21], SNN-Cliq [22]) Gaussian mixture models (TSCAN [23]) or combinations of these methods. Depending on the focus of a study, the selection of an appropriate tool is critical in terms of data set complexity, algorithm scalability, sensitivity to rare cell types, etc. One of the methods suitable for the detection of small clusters and rare cell types is DBSCAN [24], however, its efficacy in large cluster detection is limited.

The clustering was performed employing the DivIK algorithm [25]. In each iteration features were selected with the application of Gaussian mixture models for count amplitude, where features belonging to the lowest component were discarded. Next, GMM was carried out on the remaining data variances, and features belonging to the highest component were retained. The remaining features are then used for k-means clustering with the GAP criterion for determining the optimal number of clusters.

### Effect size profiling

The human sample clusters were identified with the aid of predefined cell markers, included in S2 Table. Due to the unavailability of cell type gene signatures for bovine samples, an approach based on effect size profiling was adopted. In each human and bovine sample, within every cluster, Glass rank biserial effect size coefficients were calculated [26]. Effect size metrics were calculated using the *rcompanion* R library [27]. In this way, each cluster is characterized by a vector of effect size coefficients for every gene, creating a genetic effect size profile. These effect size profiles were then used to link clusters in human and bovine samples within and between species in order to determine the bovine cell types using Spearman’s correlation analysis. Spearman’s coefficients were calculated between human clusters and bovine clusters. Clusters in bovine samples closest in correlation coefficient value to a human cell type were identified as that cell type in bovine samples.

### Effect size identification of immune responses

In the case of single samples for each condition, traditional statistical analysis of differentiation cannot be applied, due to the lack of possibility of variability assessment. Therefore, effect size ratios between treatment and control samples were used as a measure of differentiation strength. In each cell type, genes with the highest treatment to control ratio were selected as candidate biomarkers. For every sample comparison, genes with negligible effect size levels (< 0.1) in any sample were filtered out from the analysis.

## Results and discussion

### Batch effects dominate cell subtype variation when combining experiments

**Fig 2:**
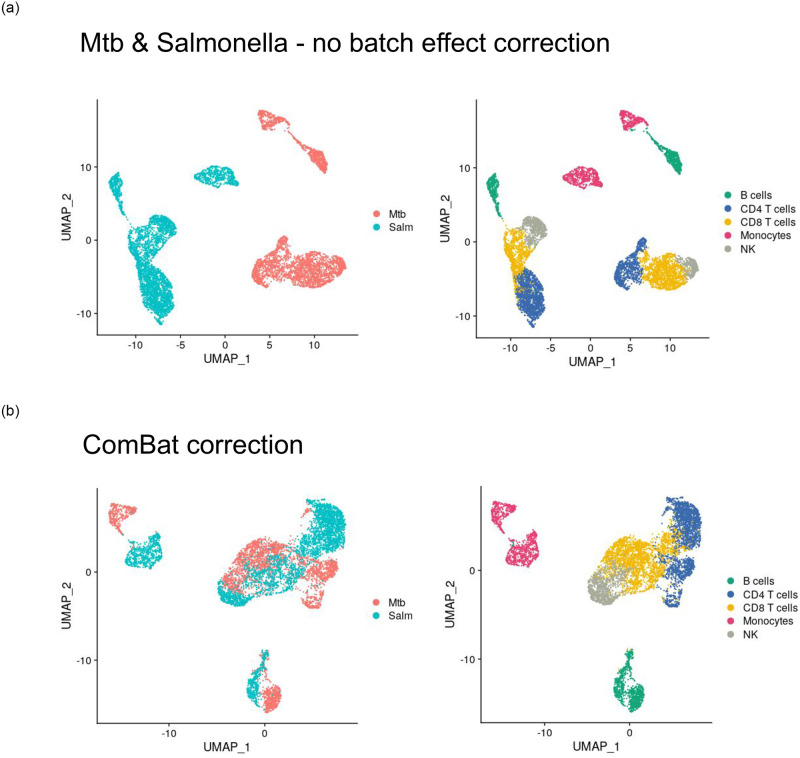
Salmonella and *M. tuberculosis* dataset UMAP projections before batch effect correction and after batch effect correction. On the left the cells are colored according to the dataset they are derived from; on the right the cells are colored according to cell type.

To evaluate batch effects upon integration of distinct experiments, we reused two scRNA-seq datasets derived from studies with human peripheral blood mononuclear cells (PBMCs). The datasets were combined with the scope of identification of cell types. The first dataset is a subset comprising control specimens and Salmonella-infected cells [28]. The second dataset consists of human naïve cells and samples infected with *Mycobacterium tuberculosis* (Mtb). Batch effects dominated cell type grouping, even after batch effect correction using ComBat (Fig 2). Despite the correction enabling clear identification of the cell clusters, ComBat was not sufficient to suppress the impact of batch effects in order to carry out statistical inference on differentially expressed genes. Moreover, the single samples available in each experiment rendered the use of batch correcting algorithms unsuitable for removing solely the unwanted bias in favor of biological variation.

**Fig 3:**
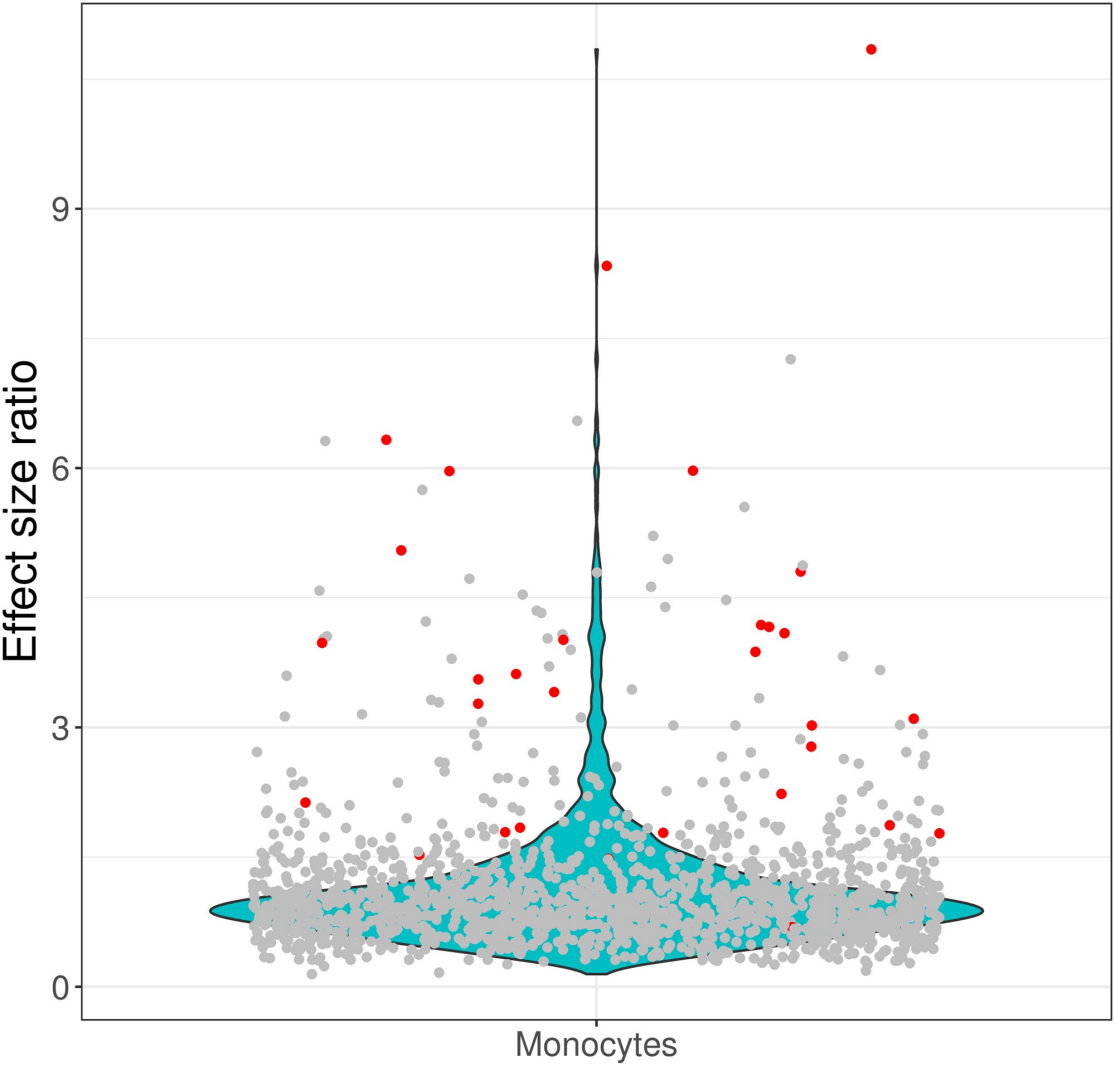
Violin plot presenting the distribution of effect size ratio values for monocytes between Salmonella stimulated and control samples. Each dot represents a gene effect size ratio value. The dots marked in red correspond to the cluster-specific genes for monocytes indicated in [28].

In view of these issues, we devised an alternative pipeline for single cell data analysis including cluster linkage and differential expression identification through effect size profiling. Glass rank biserial effect size ratios were calculated for genes between the Salmonella stimulated and control samples from the above-mentioned study [28]. The genes, where in either sample the effect size was negligible, were filtered out from the analysis. To confirm the effectiveness of detecting potential cell type biomarkers, genes presented in [28] as cluster-specific for naïve and exposed samples were assessed for the effect size ratio level in monocytes, being the cell type mainly affected by stimulation (Fig 3). The cluster-specific genes for naïve and exposed samples exhibit high levels of effect size ratios. This shows that considering effect size ratios of samples preprocessed separately is a valid approach, allowing for the identification of potential biomarkers without the need for concern with batch effects after combining samples. All the genes exhibiting high effect size ratio (above 1.5) were analysed functionally for overrepresentation in KEGG pathways. They appeared to be linked with several immune related stimulation processes, e.g. TNF signaling, Salmonella infection, NF-kappa B signaling, Cytokine-cytokine receptor interaction. The full list of enriched KEGG pathways is presented in S3 Table.

### Cluster identification in non-standard organisms through effect size profile linkage

The second issue addressed in this study is the assignment of cell type clusters in less well described organisms, based on data from another species that provides a reference in terms of a stronger established cell type structure. In this work, Divisive Intelligent K-means (DivIK) was applied as an alternative approach for clustering, which has the benefit of identifying small clusters. It is an iterative method where each time features are selected using Gaussian mixture models on the average amplitude (discarding the features in the lowest amplitude component as noise) and variance (retaining the features in the highest variance component). Then, k-means clustering is performed with the number of clusters chosen according to the GAP criterion [29]. DivIK was previously successfully applied to radiomics data [25] and an example of the transfer of the technique is presented in this paper. The data published in [30] comprise of single cell experiments concerning immune cell repertoire in mouse and naked mole-rat spleen samples. The comparison was carried out on saline treated control samples and lipopolysaccharide (LPS) stimulated animals. Weighted Dice-Sørensen indexes were calculated between clusters identified with Seurat and DivIK and are presented in Table 1. All the values of Dice indexes are close to or above 0.9, indicating strong similarity. The full confusion matrices for cluster assignments in both techniques are available in S4 Table. The clusters identified in [30] using the standard Seurat pipeline juxtaposed against the clusters detected using DivIK present a sufficient degree of similarity, while the iterative approach allows for the identification of rare cell types with a deeper exploration of the data. The original cluster assignments with a comparison to the subclusters detected using the iterative DivIK approach can be observed in S1 Fig. UMAP projections.

**Table 1:** Weighted Dice-Sørensen index (DSI) values for the comparison of cluster assignments between the original identification in [30] and the clusters determined by DivIK.

Sample	Mouse saline	Mouse LPS	Naked mole-rat saline	Naked mole-rat LPS
Weighted DSI	0.9030	0.9364	0.8815	0.9092

**Fig 4:**
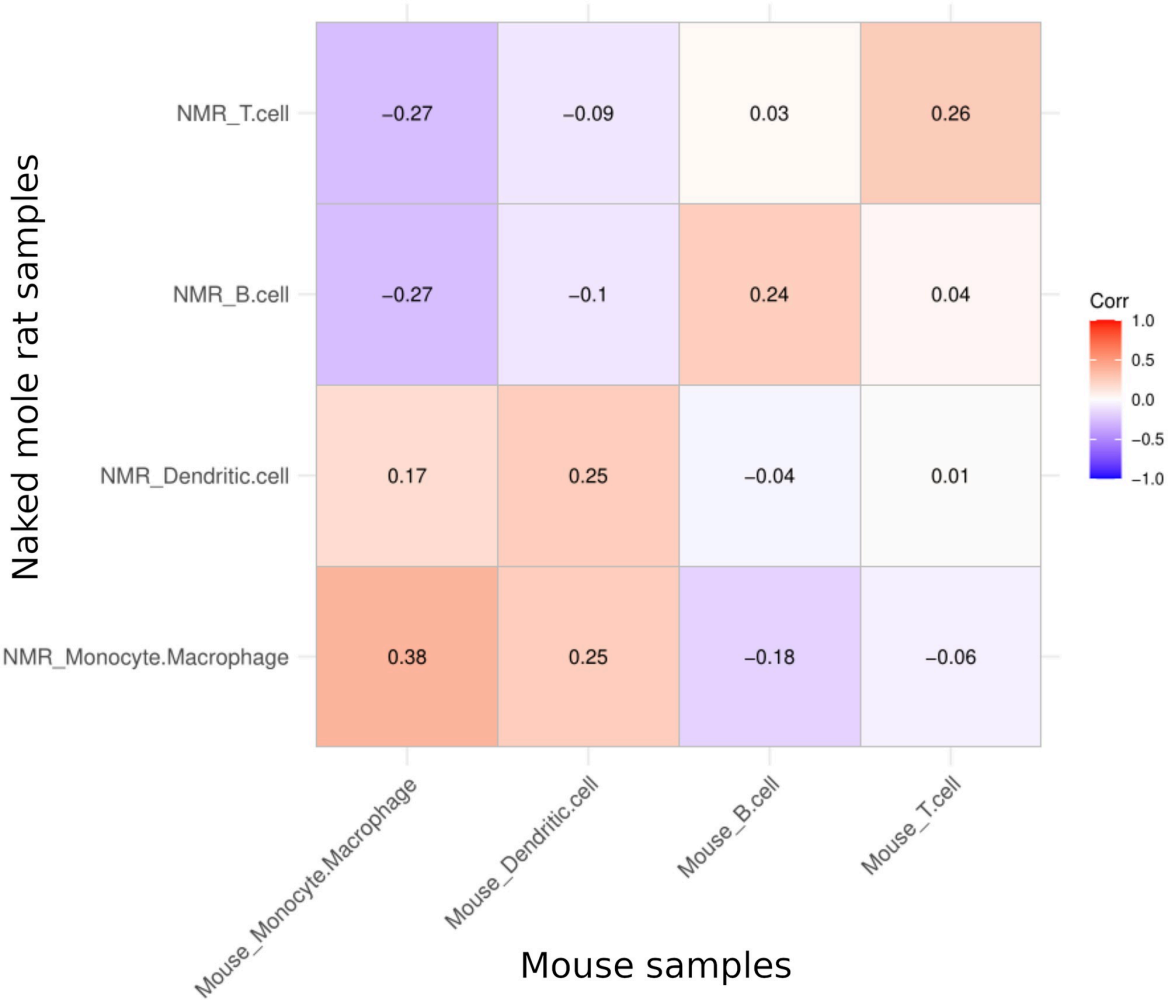
Correlogram showing Spearman’s correlation between pairs of main cell types based on their effect size profiles in the two studies species. The highest positive correlation coefficient indicates pairing cell types for naked mole rat in reference to the mouse saline treated sample.

Then, as a method for linking clusters between the naked mole-rat and the mouse, control samples treated with saline were used to assign cell types using effect size profiles. For this purpose, Glass rank biserial effect sizes were taken into consideration and Spearman’s correlation coefficients were calculated between the effect size profiles in the main cell types (T cells, B cells, Monocytes/Macrophages and Dendritic cells). Labeling of the cell types was adopted from [30]. The corresponding cell types in mouse and naked mole rat match with the highest positive correlation coefficients (Fig 4). The values of correlation coefficients are weak to moderate due to the effect size profile being calculated for the entire gene list as opposed to cell type specific genes. Nevertheless, the matching cell types confirm the technique’s efficacy, despite small correlation magnitude. In this way, the profiling based on effect size measures has been shown as an effective method for linking the cell type clusters in the naked mole-rat to the mouse cell types.

### Effect size profile identification of concordant and discordant immune responses in monocyte from various species

Following the successful use of the proposed effect size profiling and effect size-based biomarker identification approaches on the two previously published data sets, we applied the pipeline to newly generated data from a single experiment and single donor per species. The possibility of utilizing effect size-based profiles as a substitute for standard differential expression analysis was validated on a scRNA-seq data set of human and cattle PBMCs stimulated with Mtb lysates. The preprocessing filtration step led to sample sizes and feature numbers presented in Table 2.

**Table 2:** Data set sizes for human and bovine samples.

	Transcripts	Cells
Human Control	17880	4082
Human Mtb	17193	3148
Bovine Control	12160	1131
Bovine Mtb	12201	1515

The clustering analysis revealed in control human samples: CD16+ Monocytes, Monocytes, B cells, T cells, CD8+ T cells, NK cells, whereas in the Mtb samples Monocytes, B cells, CD8+ T cells, NK cells were detected. We identified in bovine samples 7 and 8 clusters in control and Mtb, respectively.

**Fig 5:**
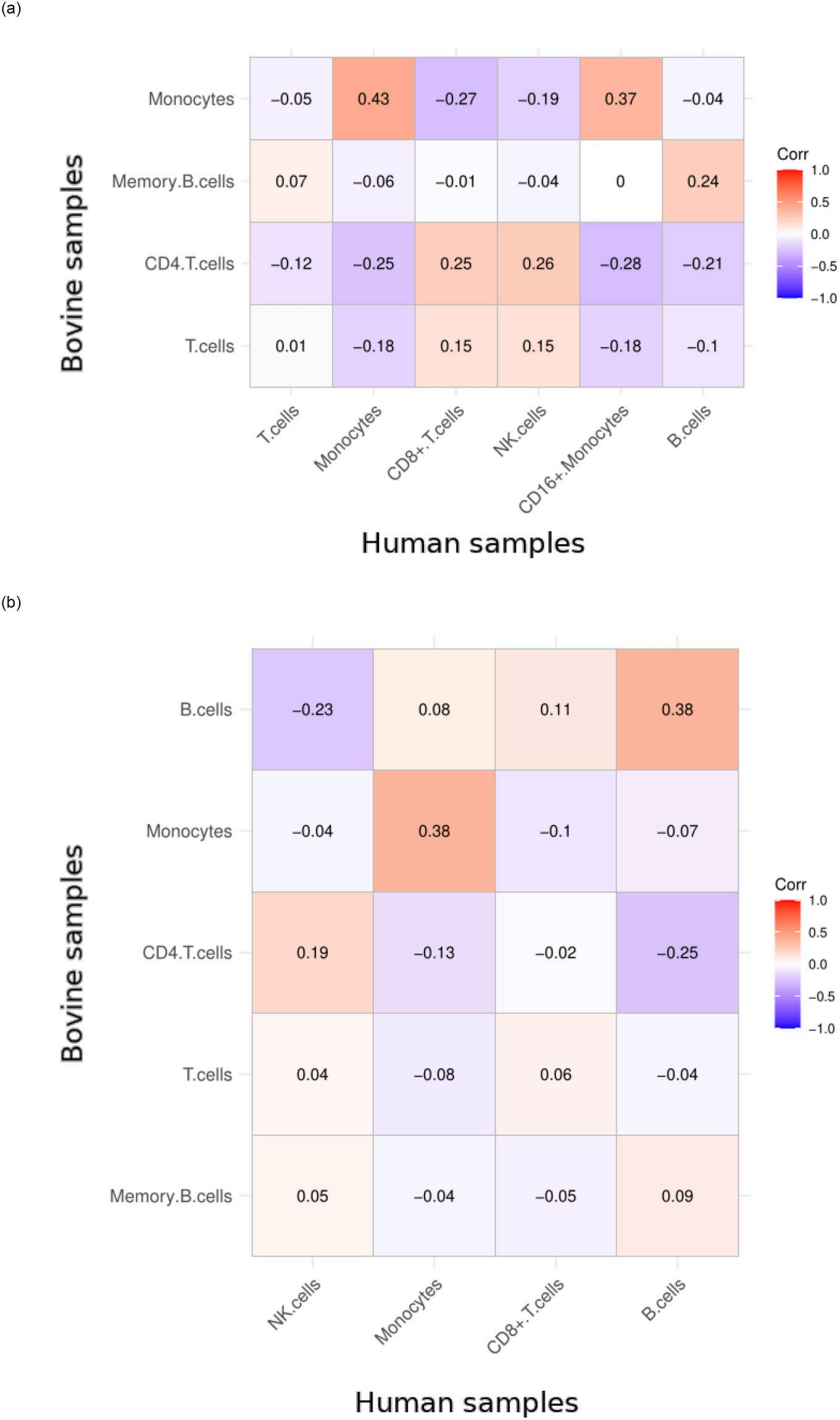
Correlograms for effect size profile vectors between human and bovine samples. The strongest positive correlation indicates a link between clusters and may serve as an indicator of homologous clusters in different species. (a) Control samples, (b) Stimulated samples.

Glass rank biserial effect size was calculated for human and bovine samples, and the effect size vectors for each cluster in the bovine samples were subjected to pairwise correlation analysis with the human sample clusters. The correlation coefficients presented linkage between clusters from both species (Fig 5).

After cell type identity was completed, effect size ratios were calculated between Mtb and control counterparts to identify differential regulated genes. The full list of effect size values and ratios is presented in S5 Table. Genes presenting effect size ratios greater than 1 in both human and bovine samples were examined in terms of their overrespresentation in KEGG pathways. The significantly enriched processes included TNF signaling, NF-kappa B signaling, Tuberculosis, IL-17 signaling. The majority of significant processes are triggered by inflammation and immune response. A full list of overrepresented pathways is provided in S6 Table. In both species, primarily monocyte clusters responded to the stimulation with Mtb lysate. As expected, genes associated with inflammation were most affected by stimulation. Effect size and differentially expressed gene analysis revealed a more robust response in the human sample compared to the bovine monocyte cluster. A subset of genes, notably PTGS2, CXCL10, TNF, IL6, IL1B, SAT1, having high Mtb to control effect size ratios and high effect size values, were chosen to be validated using qPCR.

### qPCR validation of genes identified by effect size profiling

**Fig 6:**
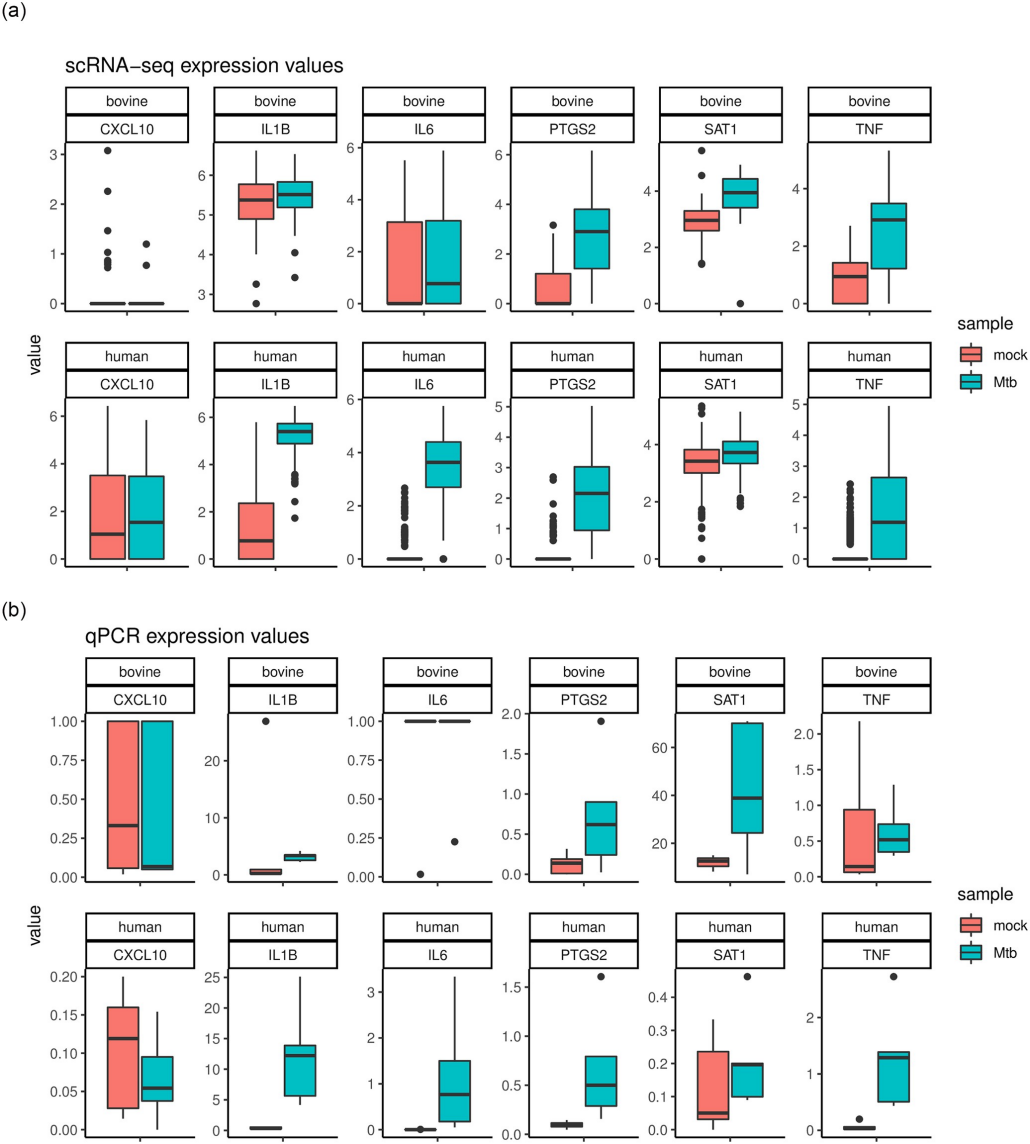
Mean expression values for genes chosen with a high effect size profile in monocytes for scRNA-seq and qPCR experiments. The control samples are marked in red, the stimulated samples in green. The immune response observed in the scRNA-seq experiments is coherent with the results of qPCR analysis.

Gene expression patterns unveiled by qPCR data were compared to expression values in the scRNA-seq data. Selected gene transcripts were quantified by qPCR: those not affected by stimulation or genes which were concordantly or discordantly upregulated in monocytes in the two species after stimulation with Mtb lysate. This was reflected by high treatment to control effect size ratios in monocytes. For this, freshly isolated PBMCs were obtained from human and cattle (n = 5) and CD14+ monocytes isolated by positive selection. In agreement with the scRNA-seq data, CXCL10 was not upregulated in either species upon stimulation with Mtb lysate, while PTGS2 and TNF were upregulated in both species. As predicted by the algorithm, SAT1 was only upregulated in bovine, but not in human monocytes. Vice versa IL6 was only upregulated in human monocytes but not in the bovine sample. Only the expression of IL1B did not match between scRNA-seq and qPCR. The qPCR experiment exposed upregulation in the human as well as bovine samples, where significant differences were not observed in bovine samples in scRNA-seq. Although, the trend seems to be concordant (upegulation under stimulation) this discrepancy may point to certain limitations of effect size profiling, namely the imperfect detection of immune process deregulation patterns in cell type specific genes. Overall, the qPCR data confirmed the predicted concordant and discordant immune responses of bovine and human monocytes detected in the scRNA-seq mean expression distributions (Fig 6).

The employment of techniques based on effect size profiles and ratios was therefore confirmed as effective not only through testing on already published data but also experimentally by validating the genes detected in scRNA-seq using qPCR.

## Conclusion

Multi-species scRNA-seq studies provide substantial information on intrinsic species-specific differences or variability in responses to stimuli, such as microbial pathogens. Such investigations are challenging given e.g., batch effects and differential expression analysis of small sample sizes. Often it is the case, that data are gathered from single experiments with single donors per experimental condition, rendering it very difficult to efficiently reduce batch effects and disabling the use of classic statistical analysis techniques for differentiation analysis. The tailored analysis steps presented herein represent a valid alternative to classic statistical testing techniques where inference based on p-values would be severely biased. Through effect size-based profiling, concordant and discordant immune responses of bovine and human monocytes have been identified in the scRNA-seq data and subsequently confirmed by qPCR validation. The limitations of the presented techniques include their design customised to situations where single samples are available and batch effects present an impossible to overcome source of bias. In cases where multiple samples are available per studied condition and batch effects are not an overwhelming component of variability, classic techniques for the analysis of deregulation patterns in single cell sequencing data will be more adequate. In conclusion, effect size profiling enables accurate analysis of scRNA-seq datasets across multiple species, and provides a chance for knowledge discovery that would be otherwise impossible to gain due to single samples and batch effects.

## Supporting information

S1 Table DivIK confusion matrices. Confusion matrices for mouse and naked-mole rat samples division into clusters between DivIK partition and the original article partition.
(DOCX)

S2 Table Cell type biomarkers. Cell type biomarker genes used to identify 15 human immune cell subtypes.
(XLSX)

S3 Table Enriched KEGG pathways for genes with high effect size ratios in monocytes between Salmonella stimulated and control samples. (XLSX)

S4 Table Mean counts, effect sizes and effect size ratios for human and bovine samples. (XLSX)

S5 Table Primer table. Primer pair, sequence base pair length and source for human and bovine target genes.
(XLSX)

S6 Table Enriched KEGG pathways for deregulated genes with high effect size ratios (greater than 1) in human and bovine samples. (XLSX)

S7 Table Validation qPCR data for selected genes human and bovine samples. (XLSX)

S1 Fig UMAP projections. UMAP projection of the original cluster assignments with a comparison to the subclusters detected using the iterative DivIK approach.
(PDF)

## References

[1] Hwang B, Lee JH, Bang D. Single-cell RNA sequencing technologies and bioinformatics pipelines. Experimental & molecular medicine. 2018;50(8):1–14. doi: 10.1038/s12276-018-0071-8 30089861 PMC6082860

[2] Kiselev VY, Andrews TS, Hemberg M. Challenges in unsupervised clustering of single-cell RNA-seq data. Nature Reviews Genetics. 2019;20(5):273–282. doi: 10.1038/s41576-019-0095-5 30617341

[3] Hicks SC, Townes FW, Teng M, Irizarry RA. Missing data and technical variability in single-cell RNA-sequencing experiments. Biostatistics. 2018;19(4):562–578. doi: 10.1093/biostatistics/kxx053 29121214 PMC6215955

[4] Tung PY, Blischak JD, Hsiao CJ, Knowles DA, Burnett JE, Pritchard JK, et al. Batch effects and the effective design of single-cell gene expression studies. Scientific Reports. 2017;7(1):39921. doi: 10.1038/srep39921 28045081 PMC5206706

[5] Butler A, Hoffman P, Smibert P, Papalexi E, Satija R. Integrating single-cell transcriptomic data across different conditions, technologies, and species. Nature Biotechnology. 2018;36(5):411–420. doi: 10.1038/nbt.4096 29608179 PMC6700744

[6] Korsunsky I, Millard N, Fan J, Slowikowski K, Zhang F, Wei K, et al. Fast, sensitive and accurate integration of single-cell data with Harmony. Nature Methods. 2019;16(12):1289–1296. doi: 10.1038/s41592-019-0619-0 31740819 PMC6884693

[7] Haghverdi L, Lun AT, Morgan MD, Marioni JC. Batch effects in single-cell RNA-sequencing data are corrected by matching mutual nearest neighbors. Nature Biotechnology. 2018;36(5):421–427. doi: 10.1038/nbt.4091 29608177 PMC6152897

[8] Smyth GK, Speed T. Normalization of cDNA microarray data. Methods. 2003;31(4):265–273. doi: 10.1016/S1046-2023(03)00155-5 14597310

[9] Leek JT, Storey JD. Capturing heterogeneity in gene expression studies by surrogate variable analysis. PLoS Genetics. 2007;3(9):e161. doi: 10.1371/journal.pgen.0030161 17907809 PMC1994707

[10] Johnson WE, Li C, Rabinovic A. Adjusting batch effects in microarray expression data using empirical Bayes methods. Biostatistics. 2007;8(1):118–127. doi: 10.1093/biostatistics/kxj037 16632515

[11] Lin Y, Ghazanfar S, Wang KY, Gagnon-Bartsch JA, Lo KK, Su X, et al. scMerge leverages factor analysis, stable expression, and pseudoreplication to merge multiple single-cell RNA-seq datasets. Proceedings of the National Academy of Sciences. 2019;116(20):9775–9784. doi: 10.1073/pnas.1820006116 31028141 PMC6525515

[12] Risso D, Perraudeau F, Gribkova S, Dudoit S, Vert JP. A general and flexible method for signal extraction from single-cell RNA-seq data. Nature Communications. 2018;9(1):284. doi: 10.1038/s41467-017-02554-5 29348443 PMC5773593

[13] Zheng GX, Terry JM, Belgrader P, Ryvkin P, Bent ZW, Wilson R, et al. Massively parallel digital transcriptional profiling of single cells. Nature communications. 2017;8(1):14049. doi: 10.1038/ncomms14049 28091601 PMC5241818

[14] Guo M, Wang H, Potter SS, Whitsett JA, Xu Y. SINCERA: a pipeline for single-cell RNA-Seq profiling analysis. PLoS Computational Biology. 2015;11(11):e1004575. doi: 10.1371/journal.pcbi.1004575 26600239 PMC4658017

[15] Zeisel A, Muñoz-Manchado AB, Codeluppi S, Lönnerberg P, La Manno G, Juréus A, et al. Cell types in the mouse cortex and hippocampus revealed by single-cell RNA-seq. Science. 2015;347(6226):1138–1142. doi: 10.1126/science.aaa1934 25700174

[16] Lin P, Troup M, Ho JW. CIDR: Ultrafast and accurate clustering through imputation for single-cell RNA-seq data. Genome Biology. 2017;18(1):1–11. doi: 10.1186/s13059-017-1188-0 28351406 PMC5371246

[17] Kiselev VY, Kirschner K, Schaub MT, Andrews T, Yiu A, Chandra T, et al. SC3: consensus clustering of single-cell RNA-seq data. Nature Methods. 2017;14(5):483–486. doi: 10.1038/nmeth.4236 28346451 PMC5410170

[18] Grün D, Lyubimova A, Kester L, Wiebrands K, Basak O, Sasaki N, et al. Single-cell messenger RNA sequencing reveals rare intestinal cell types. Nature. 2015;525(7568):251–255. doi: 10.1038/nature14966 26287467

[19] Wang B, Zhu J, Pierson E, Ramazzotti D, Batzoglou S. Visualization and analysis of single-cell RNA-seq data by kernel-based similarity learning. Nature Methods. 2017;14(4):414–416. doi: 10.1038/nmeth.4207 28263960

[20] Wolf FA, Angerer P, Theis FJ. SCANPY: large-scale single-cell gene expression data analysis. Genome Biology. 2018;19:1–5. doi: 10.1186/s13059-017-1382-0 29409532 PMC5802054

[21] Hao Y, Hao S, Andersen-Nissen E, Mauck WM, Zheng S, Butler A, et al. Integrated analysis of multimodal single-cell data. Cell. 2021;184(13):3573–3587. doi: 10.1016/j.cell.2021.04.048 34062119 PMC8238499

[22] Xu C, Su Z. Identification of cell types from single-cell transcriptomes using a novel clustering method. Bioinformatics. 2015;31(12):1974–1980. doi: 10.1093/bioinformatics/btv088 25805722 PMC6280782

[23] Ji Z, Ji H. TSCAN: Pseudo-time reconstruction and evaluation in single-cell RNA-seq analysis. Nucleic Acids Research. 2016;44(13):e117–e117. doi: 10.1093/nar/gkw430 27179027 PMC4994863

[24] Hahsler M, Piekenbrock M, Doran D. dbscan: Fast density-based clustering with R. Journal of Statistical Software. 2019;91:1–30. doi: 10.18637/jss.v091.i01

[25] Mrukwa G, Polanska J. DiviK: divisive intelligent K-means for hands-free unsupervised clustering in big biological data. BMC Bioinformatics. 2022;23(1):1–24. doi: 10.1186/s12859-022-05093-z 36503372 PMC9743550

[26] Glass GV. Note on rank biserial correlation. Educational and Psychological Measurement. 1966;26(3):623–631. doi: 10.1177/001316446602600307

[27] Mangiafico SS. rcompanion: Functions to Support Extension Education Program Evaluation; 2024. Available from: https://CRAN.R-project.org/package=rcompanion/.

[28] Bossel Ben-Moshe N, Hen-Avivi S, Levitin N, Yehezkel D, Oosting M, Joosten LA, et al. Predicting bacterial infection outcomes using single cell RNA-sequencing analysis of human immune cells. Nature Communications. 2019;10(1):3266. doi: 10.1038/s41467-019-11257-y 31332193 PMC6646406

[29] Tibshirani R, Walther G, Hastie T. Estimating the number of clusters in a data set via the gap statistic. Journal of the Royal Statistical Society: Series B (Statistical Methodology). 2001;63(2):411–423. doi: 10.1111/1467-9868.00293

[30] Hilton HG, Rubinstein ND, Janki P, Ireland AT, Bernstein N, Fong NL, et al. Single-cell transcriptomics of the naked mole-rat reveals unexpected features of mammalian immunity. PLoS Biology. 2019;17(11):e3000528. doi: 10.1371/journal.pbio.3000528 31751331 PMC6894886

