# Supplementary material for: Relative effect size-based profiles as an alternative to differentiation analysis in multi-species single-cell transcriptional studies
Source: PLoS One. 2024 Jun 25;19(6):e0305874. doi: 10.1371/journal.pone.0305874 (PMC11198858; doi:10.1371/journal.pone.0305874)
Supplement: S1 Table — Confusion matrices for mouse and naked-mole rat samples division into clusters between DivIK partition and the original article partition. (DOCX) [file pone.0305874.s001.docx]

Table X - Primer pair, sequence base pair length and source for human and bovine target genes.

| **Species** | **Target** | **Sequence (5’ 🡪 3’)** | **Base pairs** | **Source** |
| --- | --- | --- | --- | --- |
| **human** | GAPDH Fwd | CCTCAACGACCACTTTGTCA | 20 | Designed using Geneious software |
|  | GAPDH Rev | TTACTCCTTGGAGGCCATGT | 20 |  |
|  | RPS13 Fwd | CGAAAGCATCTTGAGAGGAACA | 22 | Designed using Geneious software |
|  | RPS13 Rev | TCGAGCCAAACGGTGAATC | 19 |  |
|  | CXCL10 Fwd | GTGGCATTCAAGGAGTACCTC | 21 | [1] |
|  | CXCL10 Rev | TGATGGCCTTCGATTCTGGATT | 22 |  |
|  | IL1B Fwd | CCACAGACCTTCCAGGAGAATG | 22 | OriGene Technologies, Rockville, Maryland |
|  | IL1B Rev | GTGCAGTTCAGTGATCGTACAGG | 23 |  |
|  | IL6 Fwd | CCTGAACCTTCCAAAGATGGC | 21 | [1] |
|  | IL6 Rev | TTCACCAGGCAAGTCTCCTCA | 21 |  |
|  | PTGS2 Fwd | CAATTGTCATACGACTTGCAGTGA | 24 | [2] |
|  | PTGS2 Rev | TGGCTGTGGAGCTGAAGGA | 19 |  |
|  | SAT1 Fwd | TTTATCCGTCACTCGCCGAG | 20 | [1] |
|  | SAT1 Rev | AATGCTGTGTCCTTCCGGAG | 20 |  |
|  | TNF Fwd | CCTCTCTCTAATCAGCCCTCTG | 22 | [1] |
|  | TNF Rev | GAGGACCTGGGAGTAGATGAG | 21 |  |
|  |  |  |  |  |
| **bovine** | GAPDH Fwd | GGCGTGAACCACGAGAAGTATAA | 23 | [3] |
|  | GAPDH Rev | CCCTCCACGATGCCAAAGT | 19 |  |
|  | RPS13 Fwd | CTACAAACTGGCCAAGAAAGG | 21 | Designed using Geneious software |
|  | RPS13 Rev | TCAGGGAGATCAGGAGCAAG | 20 |  |
|  | CXCL10 Fwd | AAGGGAAAGGGTGGCTCAT | 19 | Designed using Geneious software |
|  | CXCL10 Rev | AAGGCTGGGACTTAGCACATT | 21 |  |
|  | IL1B Fwd | TCCGACGAGTTTCTGTGTGA | 20 | Designed using Geneious software |
|  | IL1B Rev | TGTGAGAGGAGGTGGAGAGC | 20 |  |
|  | IL6 Fwd | TGGAGGAAAAGGACGGATGC | 20 | Designed using Geneious software |
|  | IL6 Rev | TGTTTGTGGCTGGAGTGGTT | 20 |  |
|  | PTGS2 Fwd | GGTGCCTGGTCTGATGATG | 19 | [4] |
|  | PTGS2 Rev | AGCAGCAATACGGTTCTGG | 19 |  |
|  | SAT1 Fwd | TGGAAGCAAAGAACCCCGCT | 20 | Designed using Geneious software |
|  | SAT1 Rev | TCGTGAGAAACCGCACACTG | 20 |  |
|  | TNF Fwd | CCACGTTGTAGCCGACATC | 19 | Designed using Geneious software |
|  | TNF Rev | ACCACCAGCTGGTTGTCTTC | 20 |  |

[1] A. Spandidos, X. Wang, H. Wang, and B. Seed, “PrimerBank: a resource of human and mouse PCR primer pairs for gene expression detection and quantification,” *Nucleic Acids Research*, vol. 38, no. suppl_1, pp. D792–D799, Jan. 2010, doi: 10.1093/nar/gkp1005.

[2] C. Mitchell, R. Johnson, A. Bisits, J. Hirst, and T. Zakar, “PTGS2 (Prostaglandin Endoperoxide Synthase-2) Expression in Term Human Amnion in Vivo Involves Rapid mRNA Turnover, Polymerase-II 5′-Pausing, and Glucocorticoid Transrepression,” *Endocrinology*, vol. 152, no. 5, pp. 2113–2122, Mai 2011, doi: 10.1210/en.2010-1327.

[3] C. M. Leutenegger, A. M. Alluwaimi, W. L. Smith, L. Perani, and J. S. Cullor, “Quantitation of bovine cytokine mRNA in milk cells of healthy cattle by real-time TaqMan polymerase chain reaction,” *Vet Immunol Immunopathol*, vol. 77, no. 3–4, pp. 275–287, Dec. 2000, doi: 10.1016/s0165-2427(00)00243-9.

[4] W. F. Marei, D. R. E. Abayasekara, D. C. Wathes, and A. A. Fouladi-Nashta, “Role of PTGS2-generated PGE2 during gonadotrophin-induced bovine oocyte maturation and cumulus cell expansion,” *Reproductive BioMedicine Online*, vol. 28, no. 3, pp. 388–400, Mar. 2014, doi: 10.1016/j.rbmo.2013.11.005.
