# Supplementary figures and images for: Relative effect size-based profiles as an alternative to differentiation analysis in multi-species single-cell transcriptional studies
Source: PLoS One. 2024 Jun 25;19(6):e0305874. doi: 10.1371/journal.pone.0305874 (PMC11198858; doi:10.1371/journal.pone.0305874)

# Mouse Saline

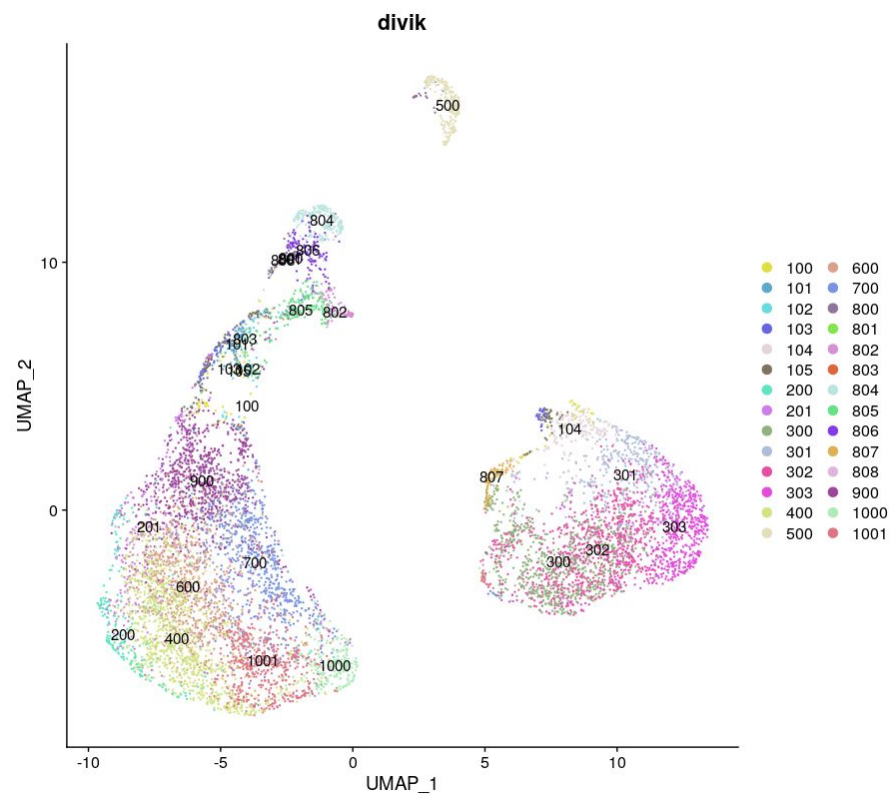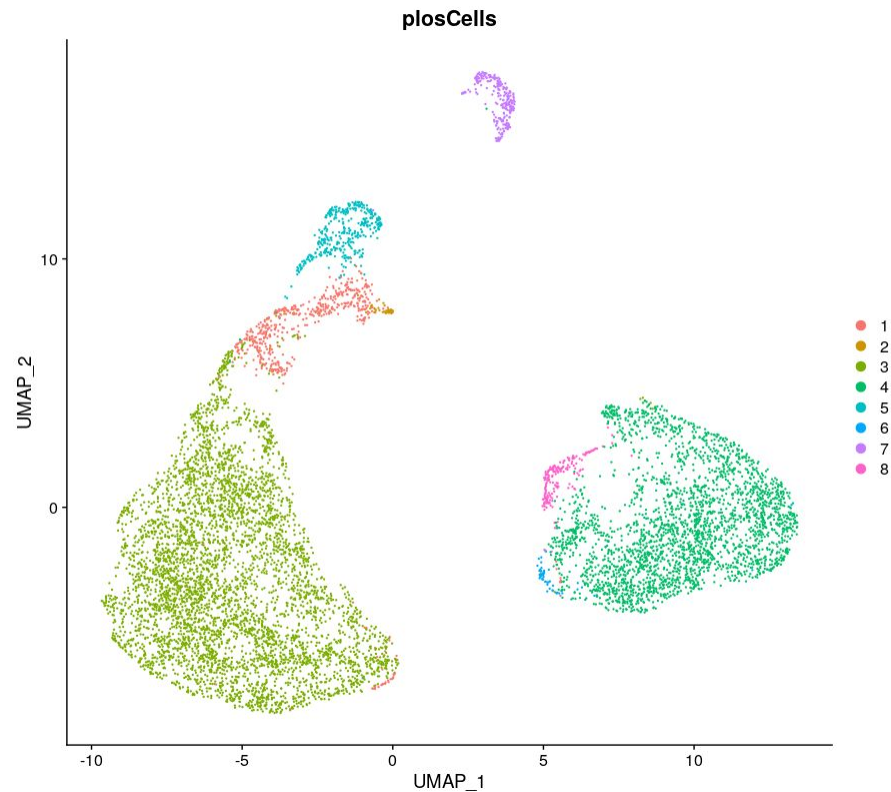

# Mouse LPS

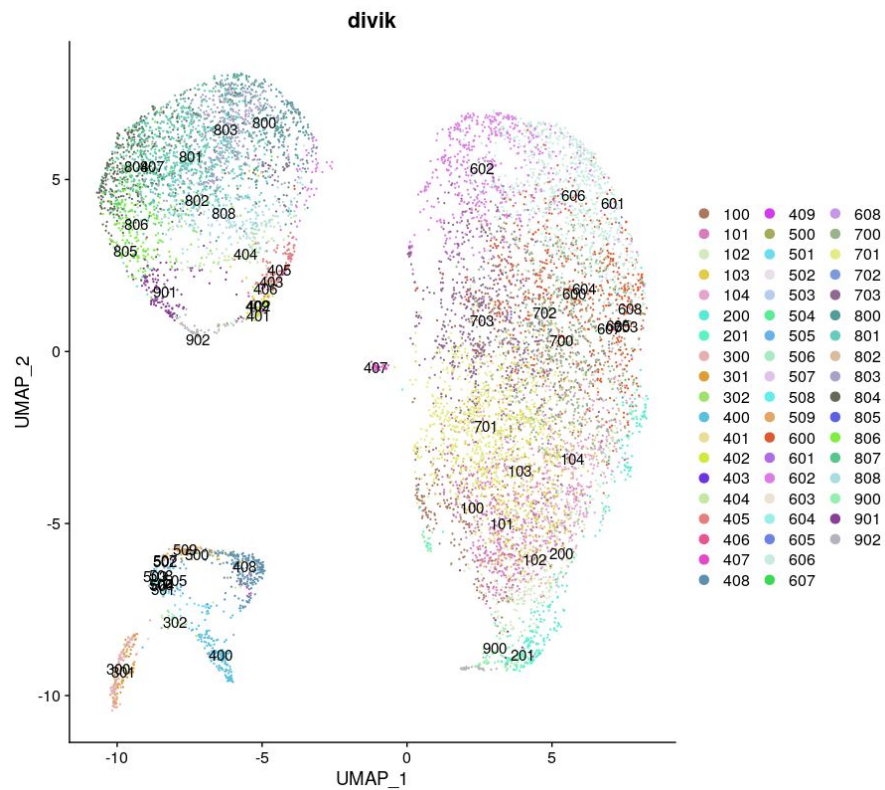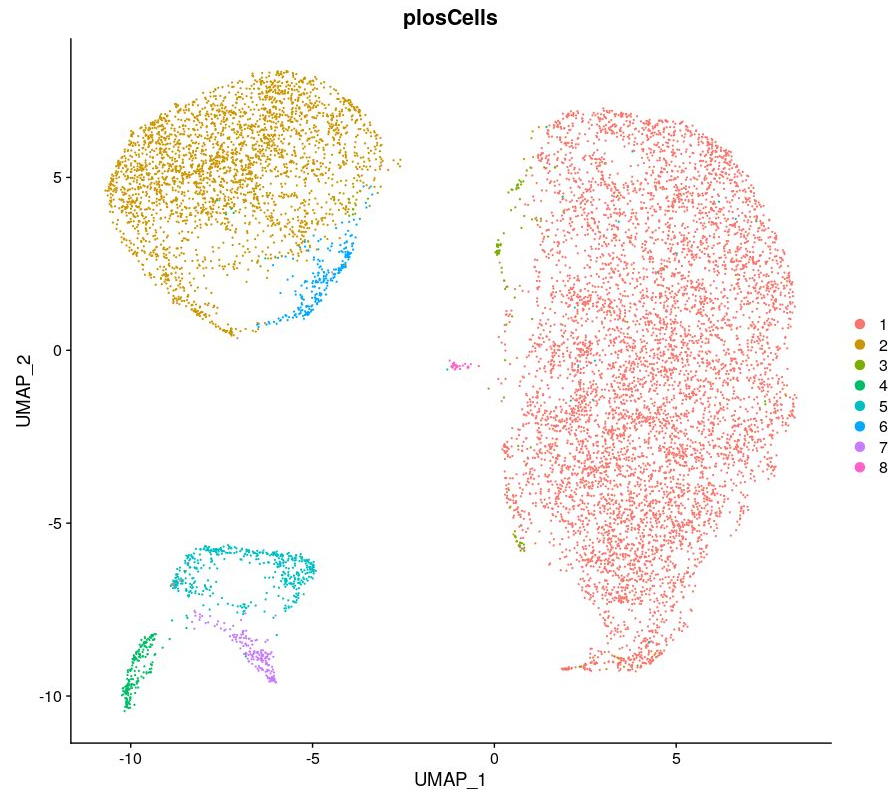

# NMR Saline

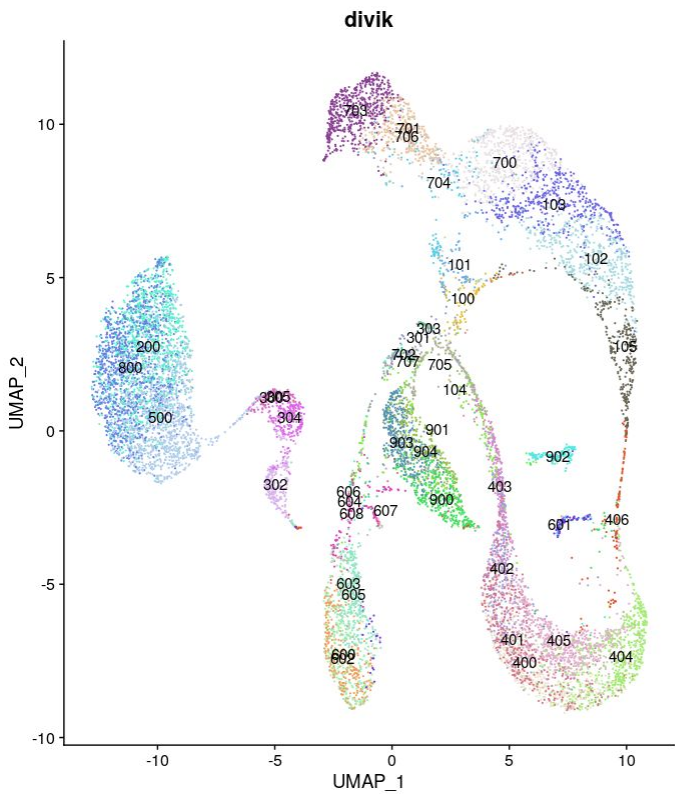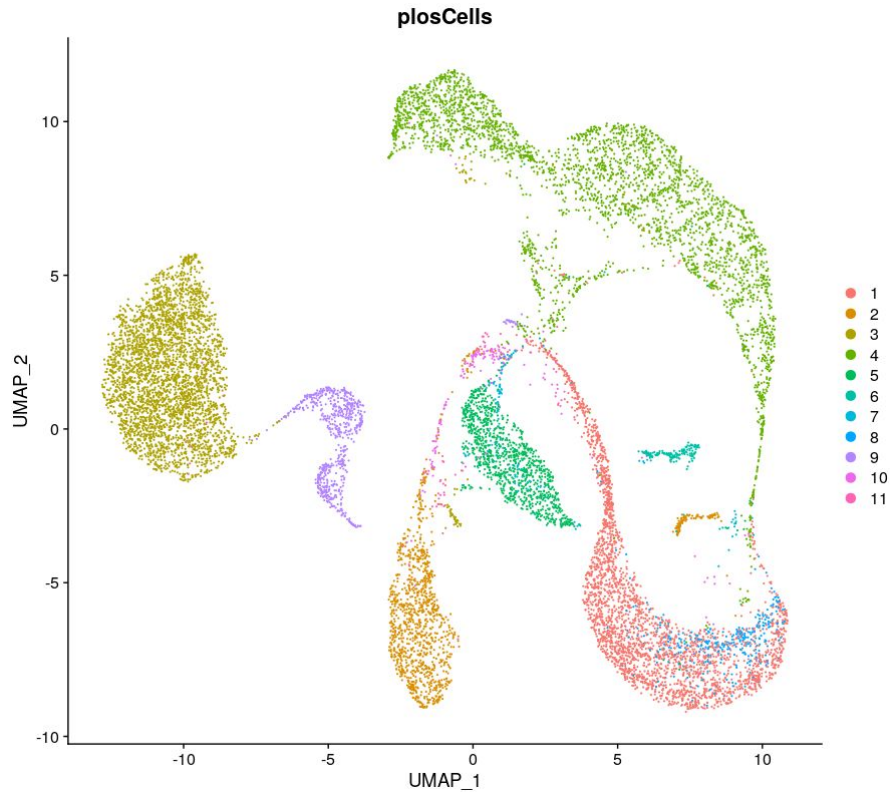

# NMR LPS

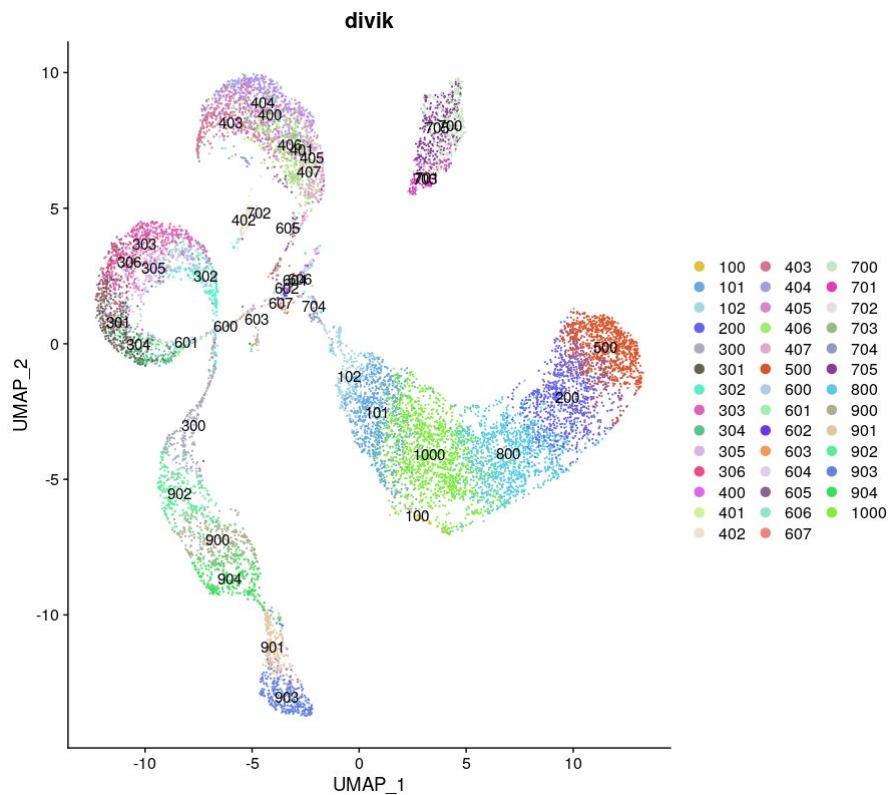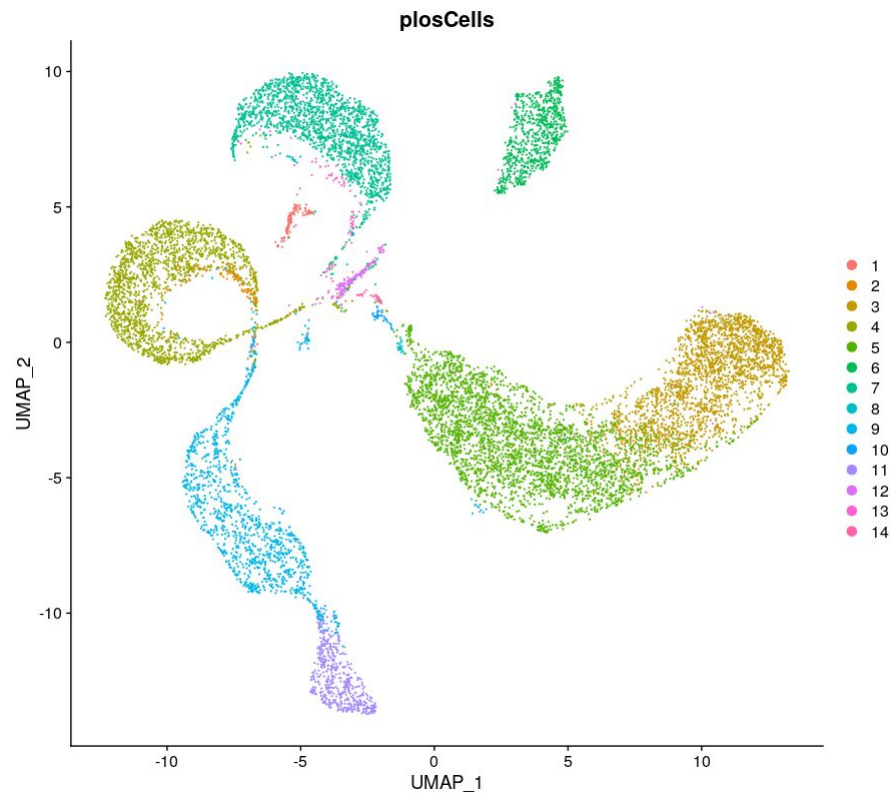

Supplement: S1 Fig — UMAP projection of the original cluster assignments with a comparison to the subclusters detected using the iterative DivIK approach. (PDF) [file pone.0305874.s008.pdf]
